# Shifts in Influenza and Respiratory Syncytial Virus Infection Patterns in Korea After the COVID-19 Pandemic Resulting From Immunity Debt: Retrospective Observational Study

**DOI:** 10.2196/68058

**Published:** 2025-07-23

**Authors:** Minah Park, Won Suk Choi, Benjamin J Cowling

**Affiliations:** 1 Department of Health Convergence, Ewha Womans University, Seoul, Republic of Korea; 2 Division of Infectious Diseases, Department of Internal Medicine, Korea University College of Medicine, Seoul, Republic of Korea; 3 WHO Collaborating Centre for Infectious Disease Epidemiology and Control, School of Public Health, Li Ka Shing Faculty of Medicine, The University of Hong Kong, G/F Patrick Manson Building (North Wing), 7 Sassoon Road, Pokfulam, Hong Kong, China (Hong Kong), 852 39176711; 4 Laboratory of Data Discovery for Health Limited, Hong Kong Science and Technology Park, New Territories, China (Hong Kong)

**Keywords:** influenza, RSV, immunity debt, epidemiology, public health

## Abstract

**Background:**

Nonpharmaceutical interventions (NPIs) such as mask-wearing and social distancing during the COVID-19 pandemic significantly reduced the transmission of common respiratory viruses, including influenza virus and respiratory syncytial virus (RSV). As NPIs were stopped, concerns emerged about “immunity debt,” which suggests that limited natural exposure to pathogens may have increased susceptibility and severity, particularly among young children. However, despite growing attention, the postpandemic impact of NPIs on epidemiologic patterns and shifts in age-specific disease burden remains underexplored.

**Objective:**

This study aims to investigate, using national surveillance data, the repercussions of the COVID-19 pandemic on the epidemiology and clinical burden of influenza virus and RSV infections in Korea, with an emphasis on the influence of NPIs on the incidence and clinical severity of these infections, particularly among young children.

**Methods:**

We analyzed weekly virologic, outpatient, and inpatient surveillance data on influenza virus and RSV infections from the Korea Disease Control and Prevention Agency from 2017 to 2024, covering the prepandemic, pandemic, and postpandemic periods. Time-series analyses were conducted to examine changes in seasonality and to estimate age-specific incidence and clinical severity of influenza virus and RSV infections before and after the COVID-19 pandemic.

**Results:**

In the postpandemic seasons, both RSV and influenza virus infections showed disrupted seasonality with delayed and prolonged epidemics. While the overall burden of both viruses was comparable to that for prepandemic periods, there was a notable shift in the age distribution of severe cases. Among influenza–associated hospital admissions, the proportion of school-aged children (7‐18 years) doubled, rising from 14% (1,814/12,660) in 2019/20 to 28% (2,176/7,755) in 2022/23. Hospitalization rates in this age group also increased significantly, from 46.8 to 64.4 per 100,000 among children aged 7‐12 years, and from 16.4 to 30.0 per 100,000 among those aged 13‐18 years. For RSV infections, the burden shifted most prominently to young children aged 1‐6 years, whose share of hospital admissions rose from 48% (5,789/11,969) to 61% (7,316/12,011) over the same period. This age group also experienced the largest rise in RSV-associated hospitalization rates, increasing from 230.8 to 357.5 per 100,000 between the 2019/20 and 2022/23 seasons.

**Conclusions:**

The patterns of influenza virus and RSV infections in Korea following the COVID-19 pandemic reveal distinct shifts in timing, severity, and the age groups that were most affected. Postpandemic influenza and RSV activity in Korea showed delayed and prolonged epidemics, with shifts in age-specific disease burden rather than an overall increase. Substantial increases in susceptibility and severity among young children for RSV infections and older children for influenza virus infections suggest lingering immunity gaps from reduced exposures during the pandemic. These effects may be further compounded by declining influenza vaccine uptake among children following the pandemic. Our findings underscore the importance of ongoing surveillance and targeted public health measures to manage respiratory viruses in the postpandemic era.

## Introduction

Nonpharmaceutical interventions (NPIs), including mask-wearing and social distancing, were widely implemented during the COVID-19 pandemic to mitigate the spread of SARS-CoV-2. While effective in controlling COVID-19, these measures also led to an unprecedented decline in the circulation of other respiratory viruses, including influenza and respiratory syncytial virus (RSV) [1-3].

The concept of “immunity debt,” which suggests that reduced exposure to common pathogens during the pandemic may have increased susceptibility and the risk of more severe outcomes upon re-exposure, has gained growing attention as an important factor affecting the postpandemic resurgence and altered epidemiology of respiratory viruses. This theory is particularly relevant to influenza virus and RSV infections [1,4-7], as early-life exposures are critical for developing immune defenses. For example, a recent modeling study demonstrated that prolonged NPIs delayed natural RSV exposure, resulting in a higher burden of infection among young children, most of whom are typically exposed to RSV by the age of 2 [8].

Numerous studies from Europe and North America have reported increased hospitalizations and shifts in age-specific severity for influenza virus and RSV infections following the COVID-19 pandemic [9-12]. A cohort study of 2809 children hospitalized with RSV in the United States found a significant increase in the proportion of children aged 2 to younger than 5 years (34.2% in 2022‐23 vs 23.4% in prepandemic seasons) [9], suggesting increased susceptibility and severity in this age group that may be linked to immunity debt. Another study of patients with RSV infections 5 years or younger in the United States reported an increase in the median age of children with RSV (11.3 mo in 2022‐23 vs 6.8 mo in prepandemic seasons) and children with severe RSV infections requiring advanced respiratory support (6.9 mo in 2022‐23 vs 4.6 mo in prepandemic seasons for high-flow nasal cannula) [10]. A cohort study in Italy also showed that RSV-associated hospitalizations significantly increased in postpandemic seasons, particularly among older age groups, whose risk of severe RSV increased by 4.7 times in 2021‐22 [11]. However, limited data are available from Asian countries that implemented more stringent and prolonged NPIs compared to many Western countries. Recent research [6] has shown that the intensity of NPIs is positively associated with the magnitude of subsequent immunity debt, with countries enforcing stricter measures experiencing larger surges in respiratory infections following the relaxation of NPIs.

As such, this study aims to fill this gap by analyzing shifts in influenza and RSV epidemiology and clinical burden before and after the COVID-19 pandemic using nationwide surveillance data in Korea, where strict NPIs, including prolonged mask mandates and rigorous social distancing policies, remained in place until as late as March 2023, and investigating how immunity debt may have manifested in this unique context.

By directly comparing influenza virus and RSV infections (often jointly discussed in the context of the “tripledemic” alongside COVID-19) across pre- and postpandemic periods, this study aims to provide a more comprehensive understanding of pandemic-driven shifts in respiratory virus patterns. Furthermore, it aims to offer novel insights into the concept of immunity debt, with a particular focus on differences in age-specific impact of influenza virus and RSV infections in the postpandemic period.

## Methods

### Study Design

We conducted a retrospective observational study to examine shifts in seasonality and age-specific incidence and severity of influenza virus and RSV infections using national surveillance data. Time-series approach was used to characterize patterns of influenza virus and RSV activities and associated hospitalization rates over the study period. Age-specific proportions of hospitalizations were compared between pre- and postpandemic, and influenza virus and RSV infections.

### Data Sources

We analyzed weekly virologic, outpatient, and inpatient surveillance data collected from September 3, 2017 (wk 36) to August 31, 2024 (wk 35) through the Influenza and Respiratory Viruses Surveillance System (KINRESS) by the Korea Disease Control and Prevention Agency (KDCA). KINRESS is a nationwide sentinel surveillance system designed to monitor influenza and other respiratory viruses, including RSV. As of February 2025, KINRESS integrates data from 300 sentinel clinics (outpatient surveillance), 18 public health laboratories in each city or province testing respiratory specimens from 106 clinics (virologic surveillance), and 221 hospitals (hospitalization surveillance) across Korea. The KDCA publishes Weekly Sentinel Surveillance Reports on its website, which are available to the public. These reports provide weekly updates on 23 Category IV infectious diseases that are considered less severe but highly prevalent, such as influenza, RSV, and other acute respiratory infections. For this study, we extracted data from these reports, including the proportion of influenza-like illness (ILI) consultations from outpatient surveillance and the proportion of respiratory specimens testing positive for influenza virus (by subtype) and RSV from virologic surveillance coordinated through the Korea Respiratory Virus Integrated Surveillance System (K-RISS), to estimate weekly influenza and RSV activity. K-RISS conducts real-time RT-PCR on respiratory specimens collected from participating clinics to test for 8 common respiratory pathogens, including influenza virus and RSV, and publishes weekly updates on the KDCA’s Weekly Sentinel Surveillance Reports. We also used the age-specific number of hospital admissions with confirmed influenza virus or RSV infections from inpatient surveillance, which is publicly available from the KDCA online portal, to estimate age-specific proportions and incidence rates of hospital admissions associated with influenza or RSV.

### Statistical Analysis

Each season was defined from week 36 of one year to week 35 of the following year, as per KINRESS. We then categorized these 7 seasons into three distinct periods as follows: prepandemic (2017/18 to 2019/20; 3 seasons), pandemic (2020/21; 1 season), and postpandemic (2021/22 to 2023/24; 3 seasons). For this study, we consider the 2021/22 season as postpandemic since it followed the government’s “Living with COVID-19” plan, a phased approach aimed at progressively lifting NPIs and returning to normal life, which began on November 1, 2021, although some control measures did continue through to mid-2022, and there was a period in early 2022 where some measures were temporarily re-introduced to manage a large surge in Omicron cases [13].

As with our previous study on influenza [14], we multiplied the proportion of patients with ILI by the proportion of respiratory specimens testing positive for each influenza type and subtype or RSV to derive a weekly indicator of influenza or RSV incidence. The proportion of patients with ILI was calculated as the percentage of total outpatient visits that meet the KDCA’s ILI case definition (ie, fever ≥38°C with cough or sore throat). The proportion of respiratory specimens testing positive for influenza or RSV was calculated based on the total number of real-time RT-PCR tests done in each week. We conducted a time-series analysis using the proxies to describe trends and seasonal patterns of influenza and RSV during the past 7 years, from 2017/18 to 2023/24. The weekly count of hospitalizations associated with influenza virus and RSV was collated by age group for each season to compare the clinical severity across pre- and postpandemic periods. We then determined influenza- and RSV-associated hospitalization rates per 100,000 persons using age-specific mid-year population estimates for 2017‐2024 retrieved from Statistics Korea [15] to adjust for demographic shifts and enable comparisons across age groups. All analyses were conducted using R version 4.1.1 (R Foundation for Statistical Computing).

### Ethical Considerations

This study used aggregated, routinely collected surveillance data that contained no personally identifiable information and was therefore granted an exemption from ethics review by the Institutional Review Board of Ewha Womans University (ref no. 2025‐0096). Individual informed consent from participants was not required.

## Results

**Figure 1.**
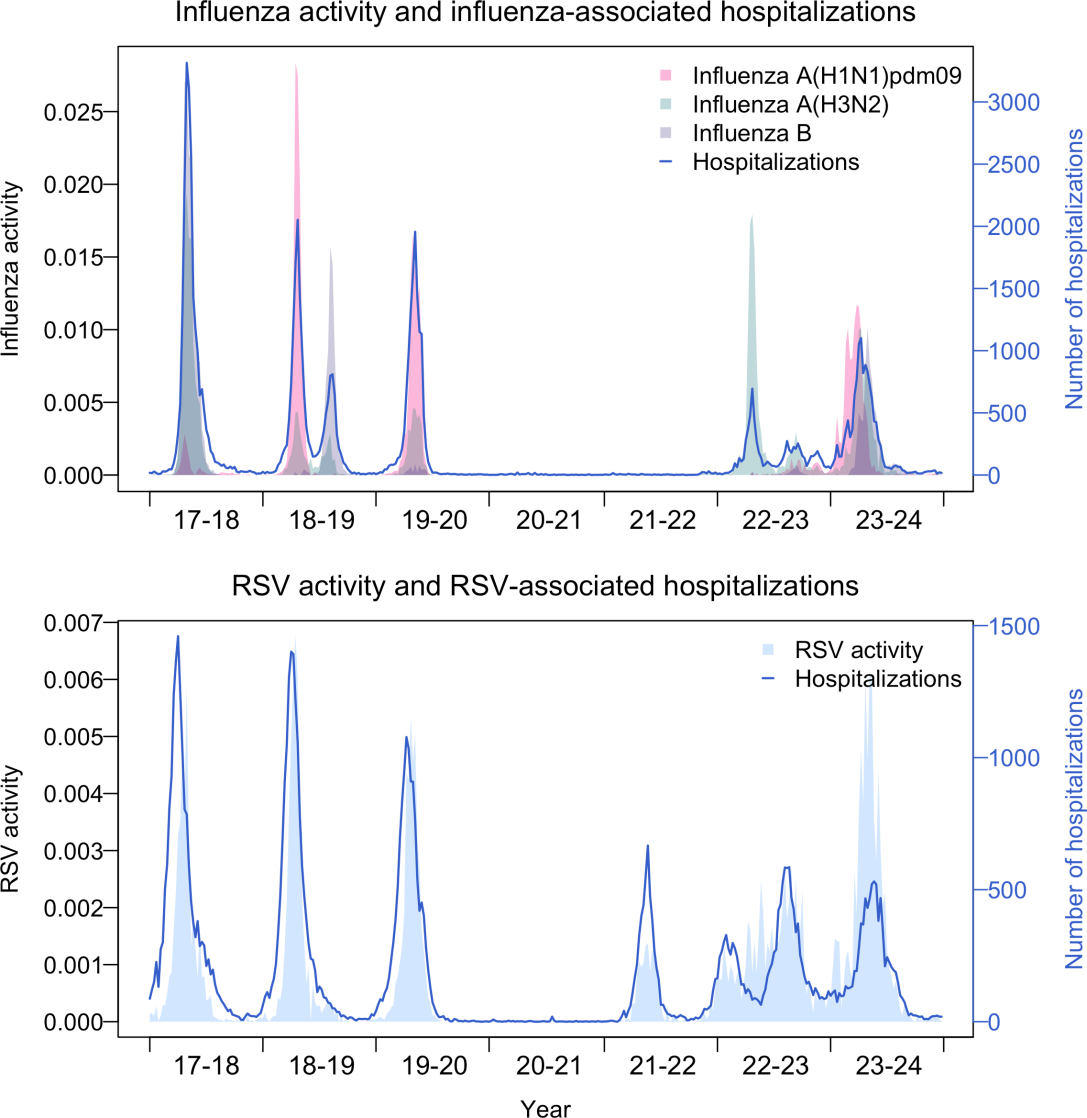
Virus activity and the number of hospitalizations: influenza and RSV in South Korea, 2017/18 to 2023/24. RSV: respiratory syncytial virus.

In prepandemic seasons, influenza virus and RSV infections followed a similar seasonal pattern, both peaking in winter, with RSV epidemics typically occurring 3‐4 weeks earlier than influenza (wk 49‐50 for RSV and 52‐2 for influenza, respectively), as illustrated in Figure 1.

Following the pandemic, RSV infections reappeared in the 2021/22 season with a delayed epidemic peak in week 5, which occurred 7‐8 weeks later than usual. In the 2022/23 season, RSV had 2 distinct peaks: an early peak in week 41, 8‐9 weeks ahead of the typical timing, and a second peak in week 17 amid a prolonged epidemic. Influenza took one more season than RSV to reappear in the 2022/23 season with a typical winter peak in week 1.

The number of influenza- and RSV-associated hospitalizations from 2017/18 to 2023/24 is shown in Multimedia Appendix 1. The overall burden was substantially reduced in postpandemic seasons. Between the 2019/20 and 2020/21 seasons, there was a significant reduction in the number of hospital admissions associated with influenza virus (n=12,660 to n=211; 98.3%) and RSV (n=11,969 to n=80; 99.3%) across all age groups (Multimedia Appendix 1). In prepandemic seasons (2017/18 to 2019/20), influenza virus and RSV infections caused a mean of 17,000 (SD 4483) and 14,583 (SD 2441) hospital admissions, respectively. In postpandemic seasons (2021/22 to 2023/24), RSV infections caused a mean of 8728 (SD 3319) hospital admissions, which is only about 60% of the admissions from prepandemic seasons.

A similar pattern was observed in influenza- and RSV-associated hospitalization rates (Table 1). The overall influenza-associated hospitalization rate significantly declined from 24.7 per 100,000 in the 2019/20 season to nearly zero in the pandemic and early postpandemic years, but bounced back to 23.9 per 100,000 by the 2023/24 season, indicating a near full recovery to prepandemic levels. As for RSV, hospitalization rates dropped significantly across all age groups during the pandemic before rebounding to near pre-pandemic levels in the 2022/23 season. The overall RSV hospitalization rate substantially declined from 23.3 per 100,000 persons to just 0.2, then to 10.5 in the 2020/21 and 2021/22 seasons, respectively. By the 2022/23 season, the rate had recovered to 23.4 per 100,000, closely mirroring prepandemic levels.

**Table 1.** Influenza- and respiratory syncytial virus–associated hospitalization rates (per 100,000 persons).

Season	Peak (wk)	Seasonal hospitalization rates (per 100,000 persons)						
		All age groups	<1 y	1‐6 y	7‐12 y	13‐18 y	19‐64 y	≥65 y
Influenza								
2017/18	1	42.2	287.5	205.6	57.5	18.6	16.3	99.4
2018/19	52	32.7	267.1	162.4	83.9	40.6	13.2	45.7
2019/20	2	24.7	189	109.5	46.8	16.4	12.0	42.9
2020/21	49	0.4	0.7	0.3	0.2	0.1	0.2	1.4
2021/22	33	0.6	1.2	1.9	0.5	0.6	0.3	1.5
2022/23	1	15.1	70.3	89.9	49.6	28.8	5.9	16.9
2023/24	50	24.3	103.8	86.6	64.4	30	9.6	49.4
RSV^a^								
2017/18	49	32.8	1811.4	275.2	8	2.5	2.2	21.3
2018/19	49	29.2	1725.5	287.6	7.8	2.6	1.5	11.4
2019/20	50	23.3	1396.2	230.8	8.4	2.2	1.6	13.4
2020/21	13	0.2	3.2	1.1	0.2	0.1	0	0.3
2021/22	5	10.5	648.1	138.0	3.5	1.8	0.4	4
2022/23	18	23.4	1236.1	357.5	10.3	3.6	1.1	9.6
2023/24	4	17.2	843.1	177.5	15.2	4.2	2.1	23.5

a RSV: respiratory syncytial virus.

**Figure 2.**
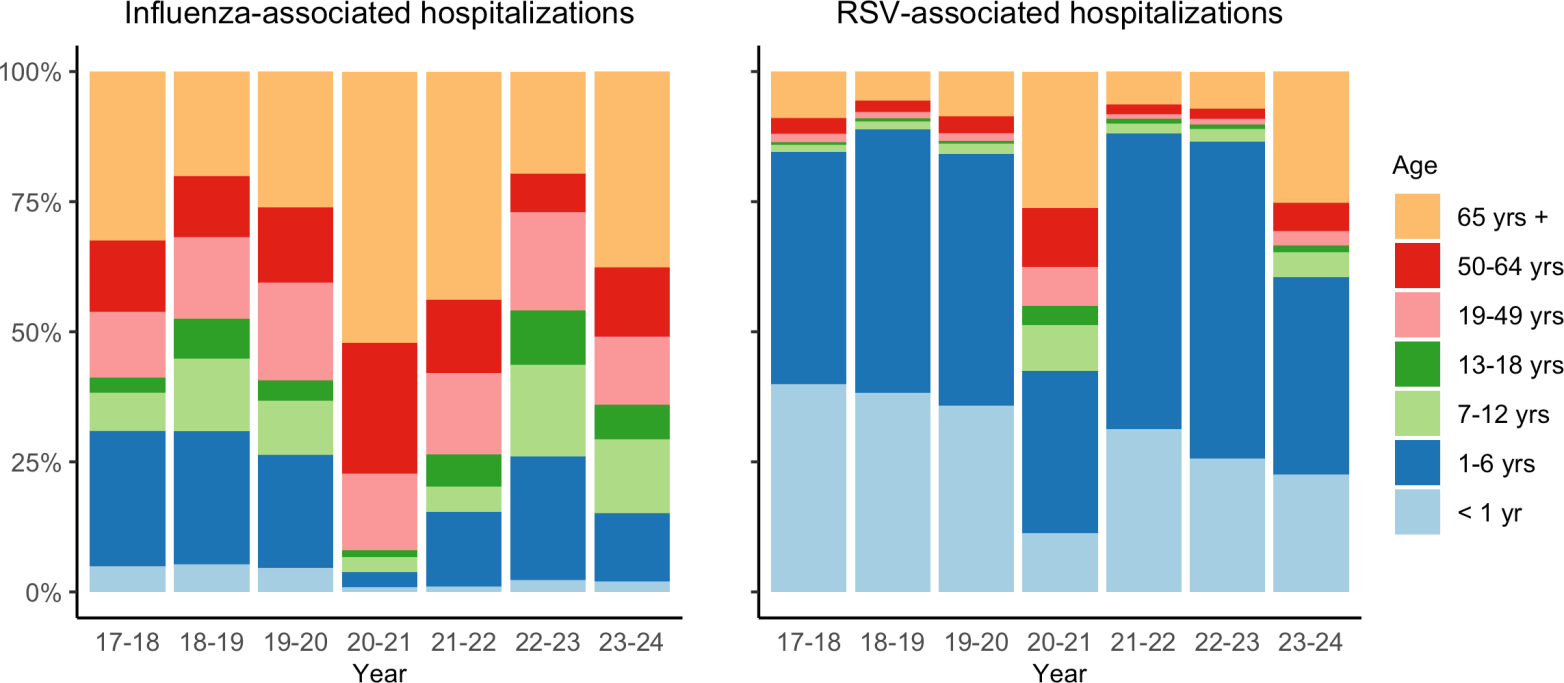
Influenza- and respiratory syncytial virus–associated hospitalizations by age group (%). RSV: respiratory syncytial virus.

For influenza virus infections, more older children aged 7‐12 years and 13‐18 years experienced severe outcomes requiring hospitalization postpandemic (Figure 2 and Multimedia Appendix 1). In the 2022/23 season, the proportion of children aged 7‐12 years was 18% (1,372/7,755), a significant increase from 10% (1,323/12,66) in the 2019/20 season. Similarly, the proportion for children aged 13‐18 years increased from 4% (491/12,660) in the 2019/20 to 10% (804/7,755) in the 2022/23 season. Similarly, children aged 7‐12 years and 13‐18 years experienced the greatest increase in influenza-associated hospitalization rates postpandemic. For children aged 7‐12 years, the rate rose from 46.8 per 100,000 in the 2019/20 season to 64.4 per 100,000 in the 2023/24 season, while for those aged 13‐18 years, it increased from 16.4 to 30.0 per 100,000. On the other hand, the hospitalization rates for infants (<1 y) and children aged 1‐6 years declined (Table 1).

For RSV infections, the proportion of young children aged 1‐6 years among RSV-associated hospitalizations increased post pandemic (Figure 2 and Multimedia Appendix 1). In the 2021/22 season, this age group accounted for 57% (3,057/5,375) of RSV hospitalizations, up from 48% (5,789/11,969) in the 2019/20 season. By the 2022/23 season, this figure rose further to 61% (7,316/12,011). This contrasts with all other age groups, whose proportions either remained the same or decreased in postpandemic seasons. Similarly, age-specific hospitalization rates show that the most significant increase in RSV-associated hospitalizations postpandemic occurred in children aged 1‐6 years (Table 1). This group saw their hospitalization rate rise from 231 per 100,000 in the 2019/20 season to 358 per 100,000 in the 2022/23 season, marking the greatest increase among all age groups. Meanwhile, infants younger than 1 year of age, who had the highest RSV burden prepandemic, experienced a considerable decline, with the rate dropping from 1400 per 100,000 in the 2019/20 season to 1,240 per 100,000 in 2022/23.

## Discussion

### Principal Findings

The widespread implementation of NPIs during the COVID-19 pandemic led to substantial reductions in the transmission of influenza virus and RSV in Korea, as it did elsewhere [1-3]. As the NPIs were progressively relaxed in late 2021, RSV returned almost immediately, followed by influenza a year later. We identified substantial changes in RSV and influenza activity, with both viruses having delayed and prolonged epidemics in postpandemic seasons. However, this prolonged activity was not linked to an increased burden. In fact, our analysis revealed a lower overall epidemiological burden compared to previous years. This was contrary to the feared “triple-demic” [16]. The lessened impact could be due to a step change in awareness of infectious disease prevention, such as more frequent hand-washing and mask-wearing after the pandemic.

An important observation in our study is the significantly increased proportion of young children aged 1‐6 years among RSV-associated hospital admissions in the postpandemic years. This may indicate that the susceptibility to severe infections, especially in young children, may be higher than in prepandemic seasons. This could be partly due to the accumulated “immunity debt,” where more young children are susceptible to severe outcomes because they have not developed immunity through prior exposures during the pandemic [4,5]. In particular, our findings suggest that immunity debt was more significant for RSV infections, particularly in children aged 1‐6 years. They were the only age group that experienced an increased severity, with an increased proportion among all RSV-related hospital admissions in the first 2 postpandemic seasons. This is consistent with a recent US study [17], which found a significantly higher RSV burden in children aged 2‐5 years, having almost 4.86 times as likely to be hospitalized in 2022 compared to prepandemic years, whereas younger children, such as infants aged 0‐5 months, were only 1.77 times as likely. Primary RSV infections tend to be more severe than re-infections [18], and by delaying primary infections for one or two years, clinical severity may have become elevated in slightly older children who had avoided RSV infection during the COVID-19 pandemic.

Our analysis revealed that the proportion of older children aged 7‐18 years increased among influenza-associated hospital admissions in the postpandemic years. It may be partly due to the delayed exposure to influenza virus infections during the pandemic, which may have resulted in reduced immunity in these school-age children. School closures, remote learning, and reduced social interactions during the pandemic likely disrupted the usual circulation of influenza, leading to fewer infections in younger populations. As restrictions eased and normal social interactions resumed, these children who had lower pre-existing immunity became more susceptible to the virus, contributing to higher hospitalization rates in this age group.

In addition, changes in vaccination coverage or the effectiveness of influenza vaccination in these years may also have played a role in the observed increase in hospitalizations. In Korea, all children aged 6 months to 13 years are currently eligible to receive free annual influenza vaccination under the National Immunization Program. While vaccination uptake among children remains relatively high compared to other countries, it has been declining in recent years, dropping from 78.5% in the 2019/20 season (prepandemic) to 72.7% in 2020/21 (pandemic), and further to 69.5% in 2023/24 (postpandemic). This slight decline in influenza vaccination coverage, combined with increased susceptibility due to immunity debt, may have contributed to the rise in influenza-associated hospitalization rates, particularly among school-aged children, who consistently have the lowest vaccination rates (82.5% for children aged 6 to 59 months of age vs 49.2% for children aged 13 years). The new RSV monoclonal antibody nirsevimab is not yet available in Korea for the prevention of RSV infections in infants.

Furthermore, while both influenza virus and RSV have returned as NPIs eased, the timing and clinical burden of these rebounds varied. RSV returning a year earlier than influenza might suggest that NPIs had less effect on RSV. RSV is thought to spread more through direct contact with contaminated surfaces and is more stable on these surfaces than influenza virus, allowing it to survive longer outside the human body [19]. This stability, combined with the virus’s prolonged shedding in infected individuals, especially children, could make RSV transmission more resilient to NPIs focused on airborne spread, such as masking and social distancing. In contrast, influenza’s shorter infectious period and reliance on airborne transmission make it more susceptible to these interventions, leading to a more effective reduction in its spread than RSV.

Our study contributes to the growing body of evidence on the postpandemic epidemiology of respiratory viruses and highlights the need for age-specific surveillance and vaccination approaches in the evolving landscape of respiratory infectious diseases. While similar trends of early and intensified RSV and influenza activity have been reported in other regions, the Korean experience offers important comparative insights due to the country’s prolonged and comprehensive implementation of NPIs. The distinct timing and magnitude of respiratory virus resurgence observed in Korea highlight how variations in public health interventions and population behaviors can differentially influence viral epidemiology. By providing data from an East Asian setting, our study expands the geographic scope of evidence on immunity debt and informs future policy planning for vaccination strategies, hospital resource allocation, and public health preparedness at the global level.

### Limitations

Our study has several limitations. First, our analyses are based on weekly virologic, outpatient, and weekly surveillance data. We did not have additional information, such as serologic data, which would allow a more comprehensive picture of the incidence and severity of infections in the community, as well as estimate population immunity. Second, changes in the burden of severe influenza virus or RSV infections in postpandemic seasons could be partially associated with increases in testing after the pandemic linked to increased laboratory capacity and attention to viral infections [20]. However, it is unlikely that increased testing fully explains our observations, particularly for RSV. In Korea, RSV diagnostic testing is generally less actively conducted than for influenza or COVID-19 due to the absence of a standard antiviral treatment, although testing is more common in infants who are at higher risk of severe illness. While increased testing postpandemic could contribute to higher reported cases, our focus on hospitalization rates rather than raw incidence mitigates this effect, as severe outcomes are less influenced by testing frequency. Furthermore, the diagnostic methods for RSV, such as rapid antigen tests, PCR, and viral culture, have not significantly changed before or after the COVID-19 pandemic. Thus, despite the pandemic’s impact on health care, the frequency and methods of RSV testing in Korea likely remained consistent, suggesting that this factor is unlikely to fully explain the observed severity shifts. This is a challenge shared by similar studies adapting to heightened postpandemic surveillance. Third, changes in health-seeking behaviors after the pandemic must also be accounted for when interpreting results from the study. The COVID-19 pandemic significantly altered how individuals accessed health care services, often delaying or avoiding visits to hospitals or clinics due to fear of exposure, public health restrictions, or overwhelmed health care systems. This shift may have resulted in the underreporting of cases in the surveillance data, affecting the comparability of pre- and postpandemic disease burden. Although altered health-seeking behaviors during the pandemic may have led to underreporting, our reliance on inpatient surveillance data, which reflects severe cases necessitating care, likely minimizes this bias. As restrictions eased post-2021, health care access likely stabilized, suggesting this limitation is more relevant to the pandemic peak than the postpandemic trends we emphasize. Future studies could refine these findings by integrating health care use data. Finally, increased awareness of respiratory infections may have heightened case detection postpandemic, but our emphasis on hospitalization trends, which should be less influenced by awareness than outpatient visits, anchors our conclusions regarding clinical severity. This awareness, potentially reducing transmission through sustained preventive behaviors, may even offset inflated detection, aligning with the lower overall burden we observed. This reflects a broader postpandemic shift in public health consciousness. Our study’s limitations, including potential biases from increased testing, shifting health-seeking behaviors, and heightened awareness, are tempered by our focus on hospitalization data and the consistency of diagnostic practices in Korea. These factors, common to postpandemic research, introduce uncertainty but do not negate the distinct shifts in influenza and RSV patterns we identified. Future analyses adjusting for these variables could further enhance our understanding of the role of immunity debt.

### Conclusions

The patterns of influenza virus and RSV infections in Korea following the COVID-19 pandemic reveal distinct shifts in timing, severity, and affected age groups. While the resurgence of RSV and influenza was anticipated with the relaxation of NPIs, the delayed and prolonged epidemic patterns observed in the postpandemic seasons did not correspond with an increased overall disease burden. Instead, we found specific increases in susceptibility among younger children for RSV infections and older children for influenza virus infections, potentially due to immunity gaps arising from limited exposure during the pandemic. These findings highlight the lasting impacts of pandemic-related disruptions on population immunity and respiratory disease transmission patterns. Ongoing surveillance and targeted public health measures remain critical to understanding and managing seasonal respiratory viruses in a postpandemic context.

## Supplementary material

10.2196/68058 Multimedia Appendix 1 Number of influenza- and respiratory syncytial virus–associated hospitalizations (n, %).

## Abbreviations

ILI: influenza-like illness
K-RISS: Korea Respiratory Virus Integrated Surveillance System
KDCA: Korea Disease Control and Prevention Agency
NPI: nonpharmaceutical intervention
RSV: respiratory syncytial virus
