# Supplementary material for: Shifts in Influenza and Respiratory Syncytial Virus Infection Patterns in Korea After the COVID-19 Pandemic Resulting From Immunity Debt: Retrospective Observational Study
Source: JMIR Public Health Surveill. 2025 Jul 23;11:e68058. doi: 10.2196/68058 (PMC12309781; doi:10.2196/68058)
Supplement: Multimedia Appendix 1 [file publichealth-v11-e68058-s001.pdf]

Supplementary Table 1. Number of influenza- and RSV-associated hospitalizations (n, %)

Season	Peak (wk)	All (N)	< 1 y	1-6 y	7-12 y	13-18 y	19-64 y	≥ 65 y
**Influenza**								
2017/18	1	21,616	1,063 (5)	5,610 (26)	1,593 (7)	626 (3)	5,700 (27)	7,023 (32)
2018/19	52	16,784	886 (5)	4,290 (26)	2,340 (14)	1292 (8)	4,599 (28)	3,377 (20)
2019–20	2	12,660	579 (5)	2,748 (22)	1,323 (10)	491 (4)	4,207 (33)	3,312 (26)
2020/21	49	211	2 (1)	6 (3)	6 (3)	3 (1)	84 (40)	110 (52)
2021/22	33	292	3 (1)	42 (14)	14 (5)	18 (6)	87 (30)	128 (44)
2022/23	1	7,755	175 (2)	1,840 (24)	1,372 (18)	804 (10)	2,040 (26)	1,524 (20)
2023/24	50	12,422	244 (2)	1,631 (13)	1,762 (14)	829 (7)	3,280 (26)	4,676 (38)
**RSV**								
2017/18	49	16,802	6,697 (40)	7,509 (45)	222 (1)	85 (1)	782 (5)	1,507 (9)
2018/19	49	14,979	5,724 (38)	7,598 (51)	217 (1)	84 (1)	512 (3)	844 (6)
2019/20	50	11,969	4,278 (36)	5,789 (48)	239 (2)	66 (1)	561 (5)	1,036 (9)
2020/21	13	80	9 (11)	25 (31)	7 (9)	3 (4)	15 (19)	21 (26)
2021/22	5	5,375	1,682 (31)	3,057 (57)	96 (2)	50 (1)	148 (3)	342 (6)
2022/23	18	12,011	3,079 (26)	7,316 (61)	286 (2)	101 (1)	368 (3)	861 (7)
2023/24	4	8,798	1,982 (23)	3,343 (38)	415 (5)	115 (1)	721 (8)	2,222 (25)
